# Clinical Determinants of Urinary Podocyte Biomarkers and Their Feasibility in Paraprotein-Related Kidney Disease

**DOI:** 10.3390/diagnostics16060922

**Published:** 2026-03-19

**Authors:** Oliver Helk, Ludwig Wagner, Gürkan Sengölge, Thomas Reiter, Daniela Gerges, Hermine Agis, Wolfgang Winnicki

**Affiliations:** 1 Department of Medicine III, Division of Nephrology and Dialysis, Medical University of Vienna, A-1090 Vienna, Austria; guerkan.sengoelge@meduniwien.ac.at (G.S.); thomas.reiter@meduniwien.ac.at (T.R.); daniela.gerges@meduniwien.ac.at (D.G.); wolfgang.winnicki@meduniwien.ac.at (W.W.); 2 Austrian Academic Institute for Clinical Nutrition, A-1090 Vienna, Austria; 3 Department of Internal Medicine III, Division of Endocrinology and Metabolism, Medical University of Vienna, A-1090 Vienna, Austria; ludwig.wagner@meduniwien.ac.at; 4 Department of Medicine I, Division of Oncology, Medical University of Vienna, A-1090 Vienna, Austria; hermine.agis@meduniwien.ac.at

**Keywords:** multiple myeloma, Paraproteinemias, podocytes, intracellular signaling peptides and proteins, biomarkers

## Abstract

**Background/Objectives:** Kidney injury is a frequent complication of multiple myeloma (MM) and monoclonal gammopathies. Podocyte stress markers, such as urinary nephrin and podocin, have been studied in other renal diseases but their utility in paraprotein-related kidney disease remains unclear. This pilot study investigated the association of urinary nephrin and podocin levels with albuminuria and biopsy-proven podocytopathy in patients with paraprotein-related diseases. **Methods:** We retrospectively analyzed 75 patients with plasma cell dyscrasias, including MM and MGRS, along with 11 healthy controls. Urinary podocin and nephrin mRNA levels were measured using qPCR, and urinary podocin protein levels were quantified via ELISA. Associations were assessed between these biomarkers and urinary protein-to-creatinine ratio (uPCR), albumin-to-creatinine ratio (uACR), and histologically confirmed podocytopathia. Diagnostic performance was evaluated using receiver operating characteristic (ROC) analysis. **Results:** Higher urinary podocin protein levels were significantly associated with lower uACR (*p* = 0.007) and uPCR (*p* = 0.026). Neither podocin nor nephrin mRNA showed significant associations with proteinuria metrics. ROC analysis indicated that podocin ELISA (AUC = 0.350) and podocin mRNA (AUC = 0.510) lacked diagnostic accuracy for predicting renal involvement. The presence of urinary tract infection (UTI) was a significant confounder, leading to increased levels of podocin and nephrin mRNA. **Conclusions:** Urinary podocin shows a trend toward elevation in MM/MGRS patients with histological podocyte injury. The study revealed an unexpected inverse association between urinary podocin and albuminuria, suggesting complex release kinetics or stage mismatches in this population. Given the confounding effect of UTIs, and the pilot nature of this study, further research is required to validate these podocyte proteins as biomarkers in paraprotein-related kidney disease.

## 1. Introduction

Kidney injury is a well-recognized complication of monoclonal gammopathies and multiple myeloma (MM) [1,2]. While tubular injury from free light chains is often the focus, along with cast nephropathy, glomerular lesions—especially podocyte injury, or podocytopathy—contribute to proteinuria and renal dysfunction in this context [3,4,5,6]. Distinguishing glomerular damage with podocyte injury from purely tubular injury has diagnostic and prognostic implications, and might allow for non-invasive longitudinal evaluation of renal response to treatment.

Podocyte-derived biomarkers detectable in urine, such as nephrin and podocin, offer a potential noninvasive window into glomerular injury [7,8,9]. Nephrin, a transmembrane protein of the slit diaphragm, plays a central role in filtration barrier integrity and in intracellular signaling, and perturbations in nephrin expression or phosphorylation are linked to podocyte dysfunction [10,11,12]. Podocin, an integral membrane protein interacting with nephrin and cytoskeletal linkers, is critical for anchoring slit diaphragm complexes and has been studied in podocyte injury models and glomerular disease [13,14,15,16]. In non-myeloma glomerular diseases, detectable nephrinuria and podocinuria have been correlated with disease activity and also with chronic histological damage [17,18]. For example, urinary nephrin has been proposed as an early indicator of glomerular injury, sometimes preceding overt proteinuria [19]. A recent clinical study also measured urinary nephrin and podocalyxin in various glomerular diseases, finding correlations with disease severity, albeit with a low sample size [17]. However, to date, there is very limited direct evidence on whether urinary nephrin or podocin levels are associated with podocytopathy in the context of MM or monoclonal gammopathy.

Given the mechanistic plausibility of noninvasive glomerular biomarkers, and the unmet need for such biomarkers in paraprotein-related kidney disease, we here investigate urinary nephrin and podocin concentrations associated with albuminuria, proteinuria and the presence of biopsy-proven podocytopathy in patients with MM or monoclonal gammopathy of renal significance (MGRS). We test this hypothesis by comparing urinary podocin and nephrin levels in patients with and without histologically confirmed podocyte injury, and by exploring their relationships with proteinuria metrics such as the albumin-to-creatinine ratio (uACR).

## 2. Materials and Methods

### 2.1. Patient Cohort

We retrospectively identified 75 patients with plasma cell dyscrasias, predominantly multiple myeloma and MGRS, along with 11 healthy controls. Frozen urine samples were available from our biobank; for a subset of patients, samples were obtained on the day of diagnostic kidney biopsy. As this work represents a pilot study focusing on processes and methods, rather than hypothesis testing or effect estimation, no detailed sample size calculation was planned. We included subjects of at least 18 years of age who had provided informed consent for sample collection and subsequent storage in our biobank. Exclusion criteria were active participation in an interventional clinical trial within the four weeks prior to sample collection. All subjects reported normal urine production and normal hydration levels at the time of sample collection.

### 2.2. Urine Samples

A total of 8 mL of morning urine was centrifuged at 3000 rpm for 10 min. The supernatant was immediately frozen at −80 °C. The urinary sediment was lysed in 1000 µL Trizol reagent.

### 2.3. RNA Isolation

The RNAzol lysate was mixed with 250 µL chloroform, then briefly mixed by frequent inverting. Phase separation was then performed by centrifugation at 12,000× *g* for 15 min. The aqueous phase was transferred to a separate tube, and RNA was precipitated by adding 500 µL isopropanol followed by centrifugation at 12,000× *g* for 20 min. After a brief wash with 70% ethanol, the RNA pellet was air-dried and resuspended in RNase-free water. The RNA was either used directly for reverse transcription or frozen at −80 °C.

### 2.4. Reverse Transcription and Real-Time Quantitative PCR

For qPCR of podocin (Hs00387817_m1) and nephrin (Hs00190446_m1), a TaqMan assay from Applied Biosystems was used. As housekeeping control, a VIC-labeled GAPDH probe set (Applied Biosystems, Foster City, CA, USA) was added to each reaction, with the water volume reduced accordingly. Gene expression levels were analyzed using the ΔΔCT method with normal kidney tissue as reference.

### 2.5. Podocin ELISA

A podocin-specific ELISA was obtained from Abcam (Human Podocin ELISA Kit, ab245708, Cambridge, UK). Urine analysis for podocin concentration was performed according to the manufacturer’s instructions.

### 2.6. Statistical Analysis

Categorical data were reported as absolute numbers and relative frequencies, and continuous data as mean ± standard deviation or median and interquartile range, as appropriate. The main continuous predictors nephrin and podocin were modeled on their original scales.

To test the null hypothesis of no difference between predictors in categorized outcome groups, we applied bootstrapped ANOVA or a *t*-test, as appropriate. To quantify associations between predictors and continuous outcomes, nonparametric Epanechnikov regression with bootstrap confidence intervals was used, as assumptions for ordinary least squares regression were not met.

Receiver operating characteristic (ROC) analyses were performed to assess the diagnostic and discriminatory performance of the assessed podocyte markers in subjects affected with a form of plasma cell dyscrasia, with the presence of albuminuria being defined as uACR ≥ 30 mg/g (positive class). Comparisons between AUCs of the different biomarkers were performed using a paired DeLong test.

Data management and analysis were conducted using R version 4.5.1 (R foundation, Vienna, Austria) and Stata 17 (Stata Corp., College Station, TX, USA). A two-sided *p*-value < 0.05 was generally considered statistically significant. Results are provided as mean ± standard deviation unless specified otherwise.

### 2.7. Ethics Approval

Approval was obtained from the local ethics committee of the Medical University of Vienna (approval number: 2435/2020)

## 3. Results

A summary of the patient cohort included in this study is presented in Table 1.

Higher levels of podocin as assessed by ELISA were significantly associated with lower amounts of urinary protein and albumin excretion (uPCR: β = −0.158, 95%CI −0.311 to 0.059, *p* = 0.026; uACR: β = −0.265, 95%CI −0.487 to 0.094, *p* = 0.007). Both podocin mRNA and nephrin mRNA showed no significant association with uPCR or uACR. (Table 2).

**Figure 1:**
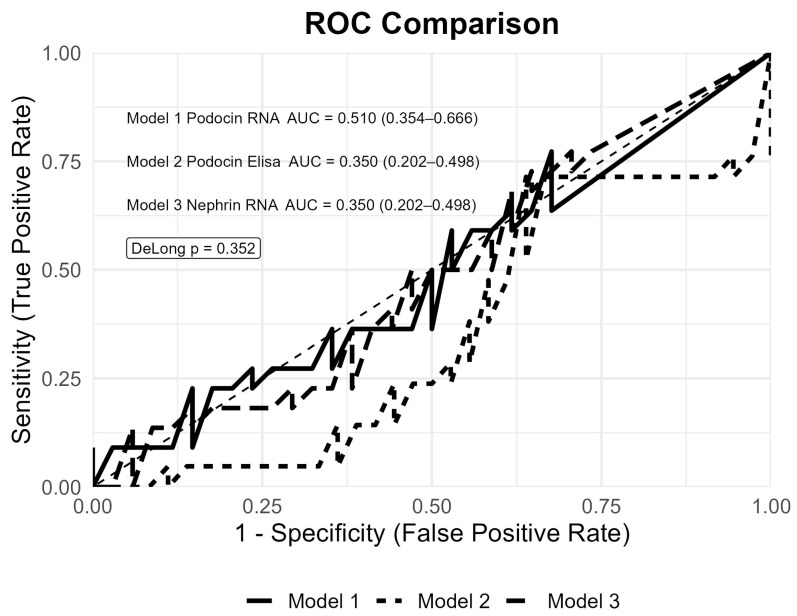
Diagnostic performance of urinary podocyte biomarkers for the presence of albuminuria in subjects with plasma cell dyscrasia and in healthy controls (n = 86).

ROC analysis, performed to evaluate the diagnostic performance of the podocyte markers in patients with plasma cell dyscrasia and albuminuria, demonstrated limited diagnostic accuracy for podocin RNA (AUC 0.510, 95%CI 0.354–0.666), podocin ELISA (AUC 0.350, 95%CI 0.202–0.498), and nephrin mRNA (AUC 0.350, 95%CI 0.202–0.498). No significant difference in predictive performance was observed between models (*p* = 0.352) (Figure 1).

Importantly, both podocin and nephrin mRNA were significantly increased when urinary tract infection (UTI) was present, while no significant differences in protein levels were shown for podocin ELISA (*p* = 0.007, *p* = 0.008 and *p* = 0.293, respectively) (Table 3). However, exclusion of the individuals with UTI did not improve the diagnostic performance of podocyte biomarkers for albuminuria (Supplementary Figure S1).

Protein levels of podocin and mRNA levels of both podocin and nephrin did not differ significantly between different forms of MGRS, MM or healthy controls (Table 4).

For mRNA and protein levels, no differences were detected in this exploratory cohort for other clinical conditions (Supplementary Table S1).

Regression analysis did not show significant association of the assessed parameters with histology confirmed podocytopathy, however, a trend towards significance was detected for podocin ELISA (β = 0.200, 95%CI 0.017 to 0.414, *p* = 0.097) (Table 5).

## 4. Discussion

In the present work we investigated whether urinary nephrin and podocin in terms of mRNA transcripts or protein are reflective of glomerular injury, as assessed by uACR levels and—where available—biopsy-proven podocytopathy, in patients with MM or monoclonal gammopathy. Our results revealed a non-significant trend toward higher urinary podocin protein levels in subjects with histologic podocyte injury. Interestingly and counter-intuitively, we also found elevated podocin to be significantly associated with lower ACR.

Although this association did not reach statistical significance, likely due to lack of statistical power as a reflection of the highly experimental nature of our study, the direction of the effect is biologically credible because podocin release into urine might reflect podocyte stress, detachment, or slit diaphragm disruption prior to or concurrent with visible glomerular injury [20]. This is in line with previous reports demonstrating podocyturia as an early marker of glomerular damage in various kidney diseases, and is in agreement with previous findings by Jiminez et al. regarding the prognostic role of urinary podocin and nephrin in diabetic kidney disease [18]. Immunoglobulin or light chain depositions along glomerular structures can trigger complement activation, and may thus preferentially affect podocytes in certain instances [21,22,23,24]. The inverse correlation between urinary podocin and ACR is counterintuitive, because albuminuria is often taken as a proxy for glomerular barrier injury [25]. Possible explanations include a temporal mismatch in disease stage, whereby podocin shedding and podocyte fragment release may precede overt glomerular albumin leakage, as well as differences in biomarker kinetics, with podocin being released early in disease progression and subsequently declining in advanced stages [26]. Thus, urinary podocin may represent a different phase of podocyte injury than ACR. Additionally, we found significantly increased levels of urinary podocin and nephrin mRNA where UTI was present, despite an absence of convincing evidence for ascending UTI or pyelonephritis. While it is possible that these markers of podocyte stress were elevated as a consequence of systemic inflammation in this context, to the best of our knowledge these findings are novel and warrant further investigation and external confirmation [27]. While these associations with current UTI, particularly when contextualized with low sample size, may explain our expected findings of ROC analysis, where podocin protein levels and nephrin mRNA levels showed worse-than-random predictive performance for renal involvement, sensitivity analysis failed to show significantly improved diagnostic performance after exclusion of subjects with UTI.

Urinary nephrin did not show robust associations in our analysis. This may reflect differences in release kinetics, because experimental models suggest that nephrin shedding occurs earlier in slit diaphragm disruption, while podocin release requires more advanced or severe podocyte detachment [28]. In conditions outside myeloma, nephrinuria correlates with severity of glomerular disease and proteinuria [17,19].

Strengths of our study include the pairing of urinary podocyte stress marker measurements with histologic confirmation of podocytopathy in a subset of subjects, which is rare in this disease domain. Limitations include a modest sample size, heterogeneity of underlying renal manifestations of monoclonal gammopathy or myeloma, a cross-sectional design, and non-availability of nephrin protein levels. Additionally, kidney biopsies were performed only in a subset of patients, as they were not deemed indicated in the remaining individuals due to a clinical lack of evidence of kidney disease.

While our results do not provide definitive answers regarding the clinical usefulness of these biomarkers, our experimental data may inform future trials evaluating the feasibility of podocyte stress markers as biomarkers in MM and MGRS.

In summary, our study provides preliminary exploratory and experimental evidence that urinary podocin may trend higher in subjects with biopsy-proven podocytopathy in MM and MGRS, and also shows an unexpected inverse association with albuminuria, while nephrin mRNA failed to show a clear relationship in this dataset. These results support further exploration of urinary podocyte proteins as noninvasive biomarkers of glomerular injury in paraprotein-related kidney disease, both in larger collectives and as components of diagnostic biomarker panels. However, our findings warrant caution when assessing podocyte markers in the presence of UTI. Alternatively, nephrin and podocin should be investigated as markers of UTI in specific investigations [29]. Future research should evaluate nephrin and podocin in larger cohorts, integrate multiple biomarkers, and assess longitudinal dynamics.

## Figures and Tables

**Table 1:** Baseline characteristics of the study cohort.

Characteristics	Total Cohort (n = 86)
Age (years)	67.2 ± 12.0
Sex—n (%)	
- male	49 (57%)
- female	37 (43%)
BMI	26.4 ± 4.9
Hematological condition—n (%)	
- MM	56 (65.1%)
- MGRS	3 (3.5%)
- LC amyloidosis	8 (9.3%)
- MGUS	8 (9.3%)
- healthy controls	11 (12.8%)
Laboratory parameters	
- uPCR (mg/g)	1016.5 ± 2551.3
- uACR (mg/g)	570.5 ± 2180.3
- eGFR (mL/min/1.73 m^2^)	63.6 ± 31.3
- serum total protein (g/L)	65.4 ± 8.8
- serum albumin (g/L)	40.2 ± 5.8
- HbA1c (%)	5.5 ± 0.5
- presence of microhematuria (%)	15 (17.9%)
Comorbidities—n (%)	
- arterial hypertension	38 (44.1%)
- type II diabetes mellitus	10 (11.8%)
- coronary heart disease	9 (10.6%)
- heart disease	3 (3.5%)
- glomerulonephritis	3 (3.5%)
- kidney transplantation	6 (7.0%)
Histomorphology—n (%)	
- number of biopsied subjects	23 (26.4%)
- evidence of podocytopathia in >20 of glomeruli	11 (47.8%)

**Table 2:** Association between urinary podocyte biomarkers with urinary protein–creatinine ratio and urinary albumin–creatinine ratio in subjects with plasma cell dyscrasia and in healthy controls.

Variable (n = 86)	β (95%CI) Podocin ELISA	*p*	β (95%CI) Podocin mRNA	*p*	β (95%CI) Nephrin mRNA	*p*
uPCR (mg/g)	−0.158 (−0.311; 0.059)	0.026	0.043 (−0.175; 0.103)	0.591	−0.045 (−0.233; 0.120)	0.643
uACR (mg/g)	−0.265 (−0.487; 0.094)	0.007	0.021 (−0.354; 0.125)	0.942	−0.026 (−0.398; 0.266)	0.878

**Table 3:** Impact of concurrent urinary tract infection (UTI) on urinary podocyte biomarkers.

	UTI Excluded	UTI Present	*p*-Value
n	67	19	
Marker			
Podocin ELISA	8.85 ± 2.33	9.00 ± 4.38	0.293
Podocin mRNA	6.56 ± 4.72	9.83 ± 4.51	0.007
Nephrin mRNA	7.05 ± 4.30	9.82 ± 3.13	0.008

**Table 4:** Comparison of urinary podocyte biomarkers across the spectrum of plasma cell dyscrasias and healthy controls.

	MM	MGRS	LC Amyloidosis	MGUS	Healthy Control	*p*-Value
n	56	3	8	8	11	
Marker						
Podocin ELISA [pg/mL]	9.22 ± 2.62	8.27 ± 1.54	6.36 ± 4.53	9.82 ± 1.63	3.92 ± 3.92	0.183
Podocin mRNA	7.27 ± 4.72	10.38 ± 1.81	8.67 ± 4.76	4.13 ± 5.19	7.67 ± 4.63	0.203
Nephrin mRNA	6.90 ± 4.63	10.70 ± 0.65	9.91 ± 1.89	6.78 ± 4.53	9.32 ± 1.54	0.141

**Table 5:** Association between urinary podocyte biomarkers and podocytopathy assessed in a subset of individuals on whom which kidney biopsies had been performed.

Variable (n = 23)	β (95%CI) Podocin ELISA	*p*-Value	β (95%CI) Podocin mRNA	*p*-Value	β (95%CI) Nephrin mRNA	*p*-Value
Podocytopathia	0.200 (0.017 to 0.414)	0.097	−0.106 (−0.396 to 0.097)	0.433	−0.334 (−1.164 to 0.430)	0.458

## Data Availability

Primary data is available upon reasonable request addressed to the corresponding author (oliver.helk@meduniwien.ac.at).

## Supplementary Materials

The following supporting information can be downloaded at: https://www.mdpi.com/article/10.3390/diagnostics16060922/s1, Table S1: *p*-value table for between-group comparisons of levels of urinary podocyte biomarkers between sexes and/or conditions. Figure S1: Sensitivity analysis: Diagnostic performance of urinary podocyte biomarkers for the presence of albuminuria in subjects with plasma cell dyscrasia and healthy controls without UTIs (n = 67).

## Abbreviations

The following abbreviations are used in this manuscript:

LC	Light Chain
uACR	Urinary Albumin-to-Creatinine Ratio
MM	Multiple Myeloma
MGUS	Monoclonal Gammopathy of Unknown Significance
MGRS	Monoclonal Gammopathy of Renal Significance
UTI	Urinary Tract Infection
