# Supplementary material for: Clinical Determinants of Urinary Podocyte Biomarkers and Their Feasibility in Paraprotein-Related Kidney Disease
Source: Diagnostics (Basel). 2026 Mar 19;16(6):922. doi: 10.3390/diagnostics16060922 (PMC13025472; doi:10.3390/diagnostics16060922)

Table S1: P-value table for between-group comparisons of levels of urinary podocyte biomarkers between sexes/conditions.

Variables (categories)	Podocin-ELISA; p-value	Podocin-mRNA; p-value	Nephrin-mRNA; p-value
Sex (male vs female)	0,783	0,236	0,101
Presence of microhematuria (yes vs no)	0,757	0,263	0,214
Arterial hypertension (yes vs no)	0,609	0,456	0,652
Diabetes mellitus (yes vs no)	0,311	0,163	0,198
Coronary artery disease (yes vs no)	0,602	0,956	0,606
Heart failure (yes vs no)	0,141	0,453	0,372

Figure S1: Sensitivity analysis: Diagnostic performance of urinary podocyte biomarkers for the presence of albuminuria in subjects with plasma cell dyscrasia and healthy controls without UTIs (n=67).

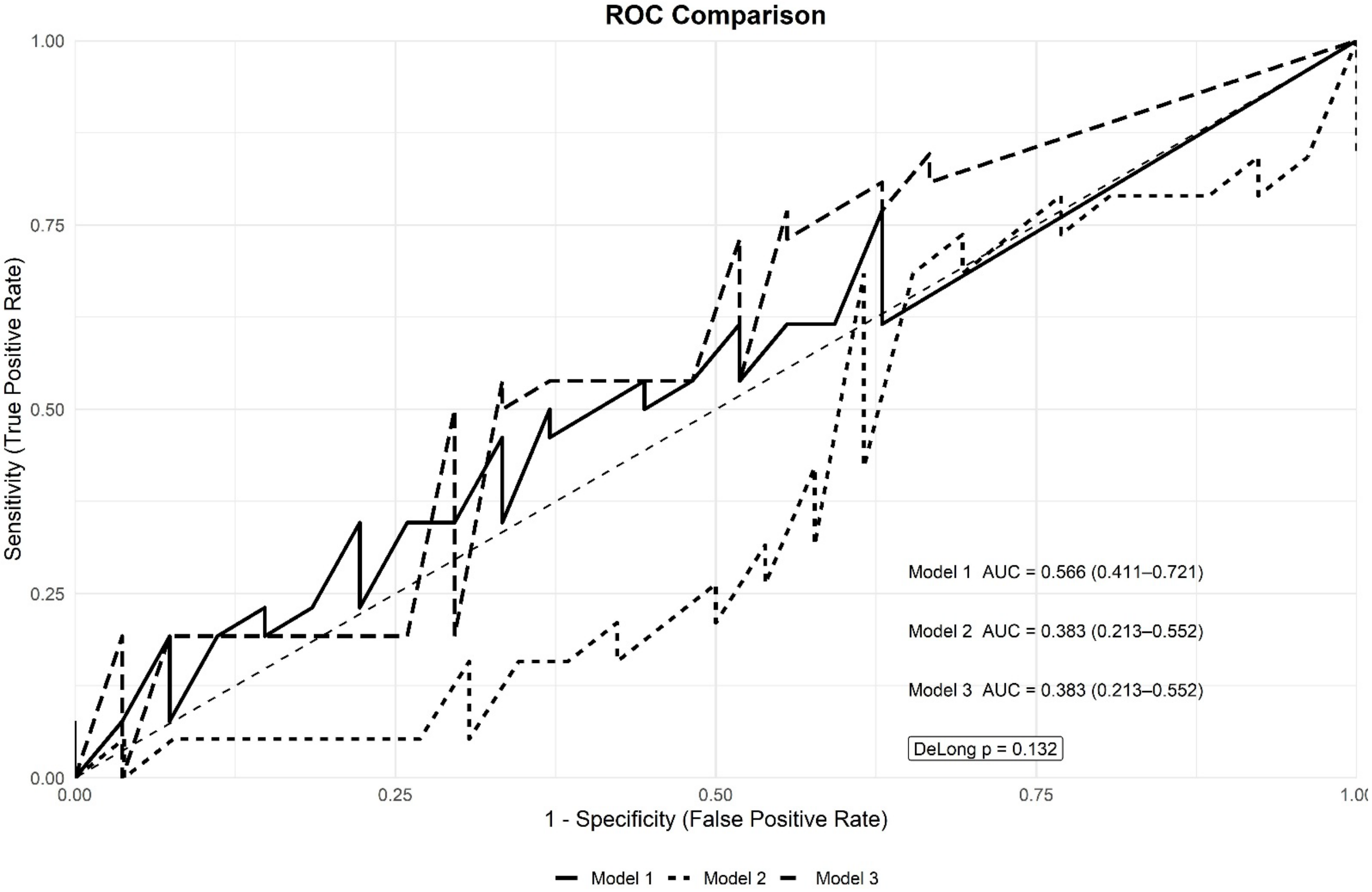

Supplement: Supplementary file 1 [file diagnostics-16-00922-s001.zip › diagnostics-4187547-supplementary (1).pdf]
